# Evaluation of Serum Zonulin Levels and Their Association with the Clinical Course of Brucellosis

**DOI:** 10.3390/life16040593

**Published:** 2026-04-01

**Authors:** Mehtap Hülya Aslan, Murat Aydın, Esra Laloğlu

**Affiliations:** 1 Microbiology Laboratory, Erzurum Faculty of Medicine, Health Sciences University, Erzurum 25240, Türkiye; 2 Infectious Diseases and Clinical Microbiology, Erzurum City Hospital, Erzurum 25240, Türkiye; kibamurat61@hotmail.com; 3 Department of Medical Biochemistry, Faculty of Medicine, Atatürk University, Erzurum 25240, Türkiye; dresralaloglu@hotmail.com

**Keywords:** acute brucellosis, chronic brucellosis, ELISA, subacute brucellosis, zonulin

## Abstract

**Background**: This study aimed to determine whether serum zonulin levels could serve as a potential diagnostic biomarker for distinguishing acute, subacute, and chronic stages of brucellosis and to evaluate their relationship with routine biochemical and hematological parameters. **Methods**: A total of 153 patients diagnosed with brucellosis and 51 healthy controls, were included in the study. Based on clinical findings, symptom duration, and serological test results, patients were classified as acute, subacute, or chronic. Serum zonulin concentrations were measured using a commercially available enzyme-linked immunosorbent assay ELISA kit (Human Zonulin ELISA Kit; BT LAB, Cat. No. E3704Hu, China). **Results**: Median zonulin levels were 24.57 (27.19) ng/mL in acute, 18.21 (14.00) ng/mL in subacute, 6.19 (2.87) ng/mL in chronic patients, and 5.81 (3.47) ng/mL in controls. Zonulin levels were significantly higher in the acute phase and decreased significantly with disease chronicity (*p* < 0.001). **Conclusions**: Serum zonulin levels were found to be higher in acute brucellosis compared to subacute and chronic cases. In brucellosis, more comprehensive research is needed to determine whether serum zonulin levels can be used as a diagnostic biomarker in the evaluation of different clinical forms.

## 1. Introduction

Brucellosis is one of the most widespread and impactful zoonotic diseases globally, caused by facultative intracellular bacteria of the genus *Brucella*. Thirteen species have been identified to date, with *B. melitensis*, *B. abortus*, *B. suis*, and *B. canis* representing the most relevant zoonotic agents due to their ability to cross species barriers, persist in animal reservoirs, and cause chronic, debilitating infections in humans [1].

Humans become infected through direct contact with infected animals such as sheep, cattle, goats, and pigs; consumption of unpasteurized milk and dairy products; ingestion of undercooked meat; or inhalation of aerosols. The disease presents with nonspecific symptoms including fever, headache, sweating, weight loss, and loss of appetite and may lead to severe complications such as endocarditis, arthritis, meningitis, and osteomyelitis. Brucellosis poses a significant threat to public health and livestock production and results in considerable economic losses worldwide [2,3].

Despite control efforts in high-income countries, brucellosis remains endemic in parts of the Middle East, Sub-Saharan Africa, South and Central Asia, and Latin America. According to the World Health Organization, brucellosis is a neglected tropical disease due to its tendency to be underdiagnosed and creates a socioeconomic burden in addition to chronic disease in humans and animals, especially dogs (*B. canis*) [4,5,6].

Owing to challenges in diagnosis and treatment, brucellosis continues to be a major public health problem associated with high morbidity [7].

The gold standard for the diagnosis of brucellosis is the isolation of the causative agent, *Brucella* spp., from blood or tissue cultures. However, the difficult growth characteristics of *Brucella*, prior antibiotic exposure, and focal organ involvement make culture-based diagnosis challenging. Consequently, serological tests are widely used in routine clinical practice. Nevertheless, serological assays may yield false-positive or false-negative results because of cross-reactivity with other infections, the presence of blocking antibodies, and variations related to different stages of the disease; this may lead to delays in diagnosis and the initiation of appropriate treatment [8]. Collectively, these challenges highlight the need for novel diagnostic biomarkers capable of reflecting the clinical course of brucellosis and supporting diagnostic evaluation.

Zonulin is a protein involved in the dynamic regulation of tight junctions between intestinal epithelial cells, thereby modulating intestinal permeability. Increased zonulin release leads to the loosening of tight junctions, facilitating the translocation of macromolecules, microorganisms, and microbial products from the intestinal lumen into systemic circulation [9]. Accordingly, elevated serum zonulin levels are considered a biochemical indicator of increased intestinal permeability [10]. Enhanced intestinal permeability has been associated with the activation of inflammatory pathways, contributing to the initiation of inflammatory cascades and acting as a biological gateway for systemic inflammation [11].

Recent studies have indicated that serum zonulin levels may increase, particularly under inflammatory and infectious conditions, and may be associated with systemic inflammatory responses [12]. However, the relationship between zonulin levels and the clinical course of brucellosis has not yet been investigated.

This study aimed to investigate whether the intense inflammatory response in brucellosis may cause transient alterations in intestinal barrier function and affect zonulin levels. Therefore, serum zonulin levels were evaluated in patients with brucellosis to determine their potential as a diagnostic biomarker for distinguishing acute, subacute, and chronic stages, as well as their relationship with routine biochemical and hematological parameters.

## 2. Materials and Methods

This single-center observational study included patients diagnosed with brucellosis who were admitted to the Infectious Diseases Outpatient Clinic of Erzurum City Hospital between 15 July 2025 and 1 November 2025. Patients were enrolled on the basis of clinical findings compatible with brucellosis and serological confirmation of the disease. In addition, a control group consisting of healthy individuals matched for age and sex was included for comparison.

The diagnosis of brucellosis was established on the basis of compatible clinical symptoms, including fever, fatigue, arthralgia, night sweats, and myalgia, and was confirmed by positive serological tests (Wright agglutination test ≥1/160 and/or Coombs anti-Brucella test ≥1/160) and/or the isolation of *Brucella* spp. from clinical specimens. Patients were classified as having acute (<2 months), subacute (2–12 months), or chronic (>12 months) brucellosis on the basis of symptom duration.

Individuals with underlying chronic diseases or significant comorbidities, pregnant women, and participants younger than 18 years were excluded from the study. Additionally, those with gastrointestinal, autoimmune, or metabolic diseases that could affect serum zonulin levels were excluded from the study. Demographic variables, including age and sex, as well as epidemiological data, such as consumption of unpasteurized dairy products, history of animal husbandry, and residence in rural areas, were recorded for all participants. In addition, hemogram results and routine biochemical laboratory parameters were retrieved from patients’ medical records.

This study was conducted in accordance with the Declaration of Helsinki, and the protocol was approved by the Health Sciences University, Erzurum Faculty of Medicine, Scientific Research Ethics Committee, decision number 2025/07-193, dated 10 July 2025.

### 2.1. Sample Size

The sample size calculation was performed via one-way analysis of variance (ANOVA), as comparisons were planned among the four groups. On the basis of Cohen’s conventional effect size criteria, an effect size of *f* = 0.27 was assumed, with a type I error rate of 5% and a statistical power of 85% [13]. Sample size, power, and effect size were revisited as simplified and practical approaches in pre-clinical, clinical, and laboratory studies [13]. The required sample size was calculated via G*Power software (version 3.1.9.7; Franz Faul, Kiel, Germany), indicating that a minimum of 44 participants per group was needed. Since the primary analysis was planned using the Kruskal–Wallis test due to the potential non-normal distribution of the data, the required sample size was adjusted according to the asymptotic relative efficiency (ARE) of the Kruskal–Wallis test relative to ANOVA. The minimum ARE value for the Kruskal–Wallis test has been reported to be approximately 0.864, indicating that about 16% more observations may be required to achieve the same statistical power as the parametric ANOVA. Therefore, the calculated sample size was increased accordingly to preserve the desired statistical power. The total sample size was determined to be 204 participants.

### 2.2. Blood Specimens

For analyses, venous blood samples were collected from the antecubital vein between 08:00 and 10:00 after an overnight fast of at least 8 h. Blood samples obtained from both the patient and control groups were allowed to clot for 30 min in an upright position at room temperature. The samples were subsequently centrifuged at 4500 rpm for 7 min at +4 °C. The separated serum samples were aliquoted and stored at −80 °C until further analysis.

### 2.3. Analyte Assay Techniques

Serum Zonulin concentrations were measured via a commercially available enzyme- linked immunosorbent assay (ELISA) kit (Human Zonulin ELISA Kit; BT LAB, Cat. No. E3704Hu, Hangzhou, China) in accordance with the manufacturer’s instructions. The ELISA test was interpreted independently by one operator who was blinded to the clinical groups. Firstly, serum samples and standards were added to microplate wells pre-coated with a human zonulin-specific antibody, allowing the zonulin present in the samples to bind to the immobilized antibodies. Samples and standards were run in duplicate. A biotinylated human zonulin antibody was subsequently added, followed by incubation with streptavidin–horseradish peroxidase (HRP). After washing to remove unbound components, a substrate mixture was added, and color development occurred in proportion to the amount of zonulin bound in each well. The enzymatic reaction was terminated by the addition of a stop solution, and the absorbance was read at 450 nm using a Rel Assay Diagnostics RL 0505 ELISA reader (Mega Tip San. ve Tic. Ltd. Şti, Gaziantep, Turkey), and results were analyzed using ELIASA-Software (version 2024.1.25). The ELISA test was interpreted independently by one operator who was blinded to the clinical groups. The analytical measurement range of the assay was 0.3–90 ng/mL, with a reported sensitivity of 0.13 ng/mL. The intra-assay and inter-assay coefficients of variation were <8% and <10%, respectively.

Alanine aminotransferase (ALT), aspartate aminotransferase (AST), and lactate dehydrogenase (LDH) levels were measured by spectrophotometric method on the Atellica Solution (Siemens Healthineers Frankfurt, Germany) device. Serum C-reactive protein (CRP) level was measured via Nephelometer method in the Siemens BN II System (Siemens Healthineers Frankfurt, Germany) device present in the biochemistry laboratory. The erythrocyte sedimentation rate (ESR) was measured using an automated Sysmex ESR analyzer (Sysmex Corp., Kobe, Japan), according to the Westergren reference method.

Complete blood count (WBC, hemoglobin, platelet count, neutrophil count, and lymphocyte count) was performed using a Sysmex XN-9000 (Sysmex Corp., Kobe, Japan) device.

### 2.4. Statistical Analysis

Statistical analyses were performed via SPSS software (version 20.0; SPSS Inc., Chicago, IL, USA). Categorical variables are expressed as numbers and percentages, whereas continuous variables are presented as medians and interquartile ranges (IQRs) or mean ± standard deviation. The normality of the data distribution was assessed via the Kolmogorov–Smirnov test. Categorical variables were analyzed via the Pearson chi-square test. Since age was normally distributed, Student’s *t*-test was used to compare two groups, the One-Way ANOVA test was used for comparisons of more than two groups, and the Post Hoc Tukey test was used to determine the significance of the difference between groups. When the assumption of normality was not met, the Kruskal–Wallis test was used, with post hoc pairwise comparisons performed via the Mann–Whitney U test where appropriate (for serum zonulin and all routine laboratory parameters). Receiver operating characteristic (ROC) curve analysis was performed to evaluate the discriminative performance of serum zonulin levels and to determine optimal cut-off values, sensitivity, and specificity. The optimal cut-off value was defined as the point with the highest Youden index (J). *p* values < 0.05 were considered statistically significant.

## 3. Results

A total of 153 patients with brucellosis and 51 healthy controls were included in the study. The mean age was 44.3 ± 15.7 years in the brucellosis group and 48.0 ± 17.1 years in the control group. There was no statistically significant difference in age between the two groups (*p* = 0.177). The sex distribution was similar between the brucellosis and control groups (*p* = 0.517) (Table 1). According to the subgroup analyses, the mean age was 44.1 ± 14.4 years in the acute brucellosis group (54.9% female), 42.6 ± 18.6 years in the subacute brucellosis group (45.1% female), 47.1 ± 14.2 years in the chronic brucellosis group (49.0% female), and 48.0 ± 17.1 years in the control group (43.1% female). No significant differences were observed among the groups with respect to the mean age (*p* = 0.288) or sex distribution (*p* = 0.618). Although unpasteurized dairy consumption, a history of animal husbandry, and rural residence were more common in the brucellosis group than in the control group, no statistically significant differences were detected (Table 1).

When routine laboratory parameters were compared among the brucellosis subgroups, significant differences were observed in the erythrocyte sedimentation rates, C-reactive protein levels, and alanine aminotransferase (ALT) levels. In contrast, no significant differences were found for aspartate aminotransferase (AST) or other hematological parameters (Table 2).

**Figure 1:**
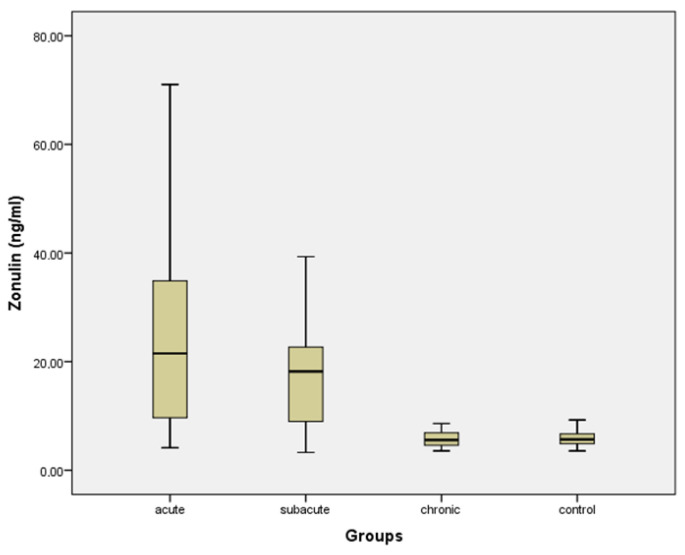
Box plot showing the serum zonulin levels in acute, subacute, and chronic brucellosis patients and healthy controls.

The serum zonulin levels were highest in the acute brucellosis group, decreased in the subacute stage, and markedly lower in the chronic stage. Zonulin levels in patients with acute brucellosis were significantly higher than those observed in both the chronic brucellosis and control groups (*p* < 0.001 for both comparisons). In contrast, no significant difference was detected between the chronic brucellosis group and the control group (*p* = 0.738) (Table 3, Figure 1).

**Figure 2:**
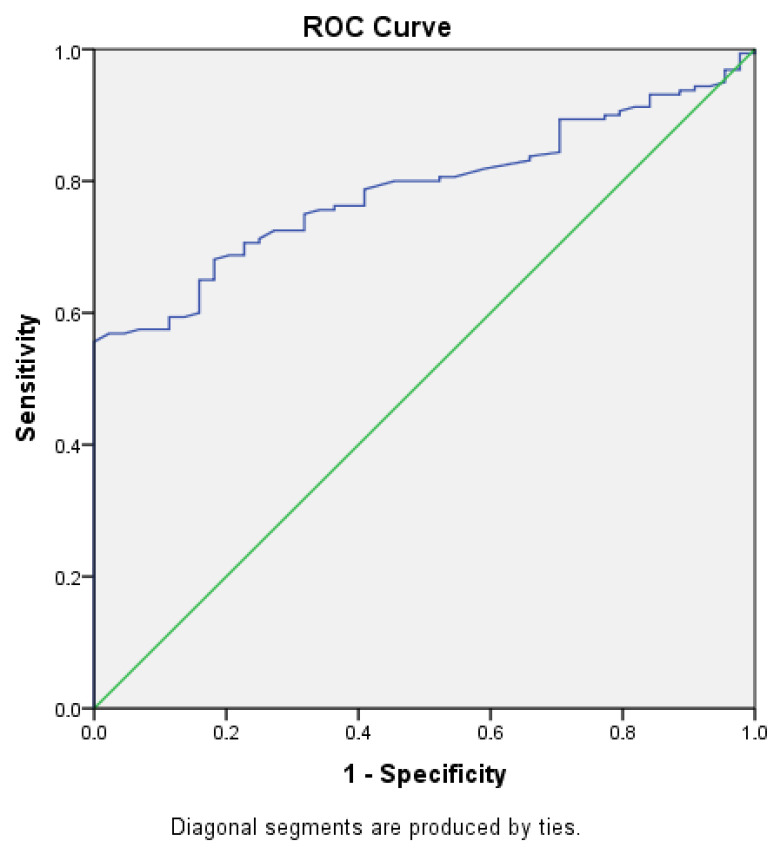
Receiver operating characteristic (ROC) curve analysis of serum zonulin levels for discriminating patients with brucellosis from healthy controls.

Serum zonulin showed a sensitivity of 81% and a specificity of 67% at a cut-off value of 9.22 ng/mL for discriminating brucellosis patients from healthy controls (AUC = 0.787; 95% CI: 0.726–0.847; and *p* < 0.001) (Figure 2).

**Figure 3:**
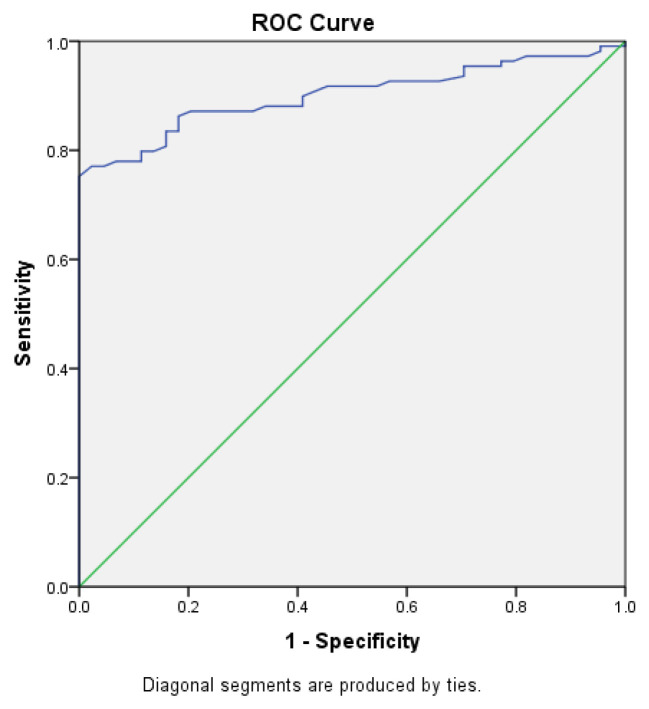
Receiver operating characteristic (ROC) curve analysis of serum zonulin levels for discriminating patients with acute–subacute brucellosis from healthy controls.

Serum zonulin showed a sensitivity of 86% and a specificity of 82% at a cut-off value of 7.42 ng/mL for discriminating acute–subacute brucellosis patients from healthy controls (AUC = 0.901; 95% CI: 0.853–0.949; and *p* < 0.001) (Figure 3).

## 4. Discussion

The gastrointestinal epithelial barrier is the largest point of contact between the external and internal environments. It provides protection against the entry of foreign antigens and microorganisms while allowing the absorption of essential nutrients, water, and electrolytes. Epithelial cells are closely linked to each other [14].

The first protein shown to have a regulatory effect on these tight junctions between intestinal epithelial cells in humans is zonulin, a haptoglobin precursor [15]. Zonulin is considered an important biomarker of high intestinal permeability [16].

Zonulin binds to two receptors: Protease-Activated Receptor 2 (PAR2) and Epidermal Growth Factor Receptor (EGFR). It then activates the phosphorylation process of myosin, a key supporting protein for TJs and Zonula Occludens-1 protein, leading to rearrangement between actin filaments and relaxation of tight junction proteins. As a result of this mechanism, permeability of the intestinal epithelial barrier increases, and the passage of antigens from the lumen into the systemic circulation is facilitated [17,18].

This molecular mechanism suggests that the systemic inflammatory response observed in acute brucellosis may contribute to an increase in serum zonulin levels by disrupting intestinal barrier integrity.

Increased zonulin levels indicate that intestinal barrier integrity is compromised and the paracellular pathway is opened [19]. Disruption of the intestinal barrier prepares the ground for a number of pathological conditions such as dysbiosis, the transport of microorganisms and microbial products across the mucosal barrier to deeper tissues, activation of the immune system, and the development of chronic inflammatory processes [14]. Increased intestinal permeability allows for the passage of microbial products, such as lipopolysaccharides (LPS) located in the outer membrane of Gram-negative bacteria, from the intestinal lumen into systemic circulation. These microbial products contribute to the increased release of pro-inflammatory cytokines and strengthening of the systemic inflammatory response by activating the immune system [20].

Brucellosis-causing *Brucella* spp. are facultative intracellular bacteria characterized by their ability to modulate the host immune response [21,22]. The cellular immune response that develops during the acute infection period is particularly characterized by Th1-mediated cytokine release; this leads to a more pronounced systemic inflammatory response [23].

*Brucella* spp. persists within host cells and causes chronic infections through various immune evasion mechanisms. It modifies its lipopolysaccharides (LPS) to reduce TLR4-mediated immune recognition and prevent the activation of pro-inflammatory pathways. After entering macrophages, Brucella avoids lysosomal fusion and proliferates in specialized vacuoles [4]. For these reasons, the diagnosis of Brucella infections, which tend to recur and become chronic, is based on clinical findings along with positive serological tests or cultures. However, serological tests remain positive for a long time after treatment and may therefore be insufficient for evaluating the response to treatment. Furthermore, they are not useful for differentiating between acute, subacute, chronic, and recurrent infections [24].

In our study, serum zonulin levels were compared in patients with acute, subacute, and chronic brucellosis, and it was shown that zonulin levels significantly increased during the acute phase of the disease and gradually decreased as the disease progressed to the subacute and especially chronic phases, returning levels similar to that of the healthy control group. In this respect, zonulin can be considered a potential diagnostic biomarker that can contribute to differentiating the clinical stages of brucellosis. These findings that we have reported are quite important, because they indicates, for the first time in the literature, that zonulin levels are evaluated together with the clinical stages of brucellosis.

The fact that zonulin levels were found to be low in chronic brucellosis cases in our study, similar to the control group, may be related to the suppression of the inflammatory response in chronic infections, the development of immune tolerance, and the formation of an adaptive rearrangement in the intestinal barrier. Brucella’s immune evasion mechanisms and its capacity to incite low-grade inflammation may have contributed to the decreased zonulin release. This finding suggests that zonulin may be a biomarker reflecting not only the presence of inflammation but also the clinical stage of the infection. In cases where serological tests are insufficient to differentiate the stage of the disease, serum zonulin levels are thought to be helpful in distinguishing acute brucellosis from subacute and chronic forms.

When these data are evaluated together, it is thought that the high zonulin levels detected in patients with acute brucellosis in our study may be a reflection of the inflammatory response accompanying the infection. Furthermore, the high zonulin levels detected in patients with acute brucellosis may be a consequence of this intense inflammatory response leading to transient changes in intestinal barrier function.

Serum zonulin levels have been investigated in various inflammatory and metabolic diseases as a marker associated with increased intestinal permeability. In their studies, Tonyalı NV. et al., Jayashree, B. et al., Oledzki S. et al., and Gürel RH. et al. found high serum zonulin levels, similar to our study, and reported that it could be used as a potential diagnostic biomarker [9,20,25,26].

The relationship between serum zonulin levels and intestinal permeability has also been identified in various diseases including inflammatory bowel diseases, celiac disease, allergic asthma, and obsessive–compulsive disorder [27,28,29,30].

### Limitations

This study has several limitations. First, its single-center design and relatively limited sample size may restrict the generalizability of the findings. Second, the lack of concurrent evaluation of zonulin levels with other established markers of intestinal permeability represents an important limitation of the study. In addition, the absence of longitudinal monitoring of serum zonulin levels after treatment limits the assessment of their potential prognostic value.

## 5. Conclusions

In conclusion, this study demonstrates that zonulin reflects increased intestinal permeability in acute brucellosis and may be a potential diagnostic biomarker in differentiating clinical stages of brucellosis. However, to differentiate brucellosis stages, clarify the clinical significance of zonulin as a biomarker, and evaluate its prognostic value for treatment response, studies in larger patient series and prospective designs are needed.

## Figures and Tables

**Table 1:** Demographic and epidemiological characteristics of patients with brucellosis and the control group.

Variable	Brucellosis Group (*n* = 153)	Control Group (*n* = 51)	*p* Value
Female, *n* (%) Male, *n* (%)	76 (49.7%) 77 (50.3%)	22 (43.1%) 29 (56.9%)	0.517
Mean age ± SD (years)	44.3 ± 15.7	48.04 ± 17.14	0.177
Consumption of unpasteurized dairy products, *n* (%)	130 (85.0%)	38 (74.5%)	0.138
Animal husbandry, *n* (%)	98 (64.1%)	29 (56.9%)	0.453
Living in rural areas, *n* (%)	106 (69.3%)	34 (66.7%)	0.862

**Table 2:** Comparison of routine laboratory parameters among patients with acute, subacute, and chronic brucellosis.

Laboratory Parameters	Acute Brucellosis (*n* = 51)	Subacute Brucellosis (*n* = 51)	Chronic Brucellosis (*n* = 51)	*p* Value
WBC (×10^3^/mm^3^)	7.00 (2.92)	6.54 (2.67)	6.68 (2.22)	0.589
Hemoglobin (g/dL)	14.80 (1.73)	14.70 (1.85)	14.90 (2.85)	0.316
Platelet count (10^3^/mm^3^)	279 (109)	261 (115)	252.5 (68.8)	0.810
Neutrophil count (10^3^/mm^3^)	3.93 (2.32)	3.67 (2.81)	3.77 (2.06)	0.725
Lymphocyte count (10^3^/mm^3^)	2.20 (1.2)	1.96 (1.13)	1.97 (0.79)	0.629
Sedimentation rate (mm/h)	8 (20)	6.5 (12.8)	4 (7)	0.016 ^a^
CRP (mg/L)	14.9 (30.9)	5 (6.7)	3 (2)	<0.001 ^a,b^
ALT (U/L)	30.5 (31.0)	25 (11.5)	25 (11.3)	0.042
AST (U/L)	21 (15.5)	20 (11)	18 (10.2)	0.064
LDH (U/L)	218 (140)	218.5 (83.5)	206 (44)	0.513

Values are presented as median (interquartile range, IQR). ^a^ Comparison between the acute and chronic groups; ^b^ comparison between the acute and subacute groups.

**Table 3:** Comparison of serum zonulin levels among the study groups.

Group	*n*	Zonulin (ng/mL), Median (IQR)
Acute brucellosis	51	24.57 (27.19)
Subacute brucellosis	51	18.21 (14.00)
Chronic brucellosis	51	6.19 (2.87)
Control group	51	5.81 (3.47)

Statistical comparisons: Acute vs. subacute: *p* = 0.27; acute vs. chronic: *p* < 0.001; acute vs. control: *p* < 0.001; subacute vs. chronic: *p* < 0.001; subacute vs. control: *p* < 0.001; and chronic vs. control: *p* = 0.738.

## Data Availability

The original contributions presented in this study are included in the article. Further inquiries can be directed to the corresponding author.

## Abbreviations

ALT	Alanine aminotransferase
AST	Aspartate aminotransferase
BCV	Brucella-containing vacuole
CRP	C-reactive protein
EGFR	Epidermal growth factor receptor
ELISA	Enzyme-linked immunosorbent assay
HRP	Streptavidin-horseradish peroxidase
LDH	Lactate dehydrogenase
LPS	Lipopolysaccharide
OD	Optical density
PAR2	Protease-activated receptor-2
ROC	Receiver-operating characteristic curve
TJ	Tight junctions
WBC	White blood cell count
